# Supplementary material for: Nuclear energy acceptance in Poland: From societal attitudes to effective policy strategies—Network modeling approach
Source: PLoS One. 2024 Aug 2;19(8):e0305115. doi: 10.1371/journal.pone.0305115 (PMC11296647; doi:10.1371/journal.pone.0305115)
Supplement: S1 File — The supplementary materials include: Dataset: This contains the raw data used in the analysis. Supplementary explanations: This document provides a breakdown of the code and additional explanations related to the model used in the analysis. Supplementary tables: This document contains supplementary tables with additional data and information related to Figs 2 and 3 in the main text. R script: This is the R script containing the code used for data analysis and generating the results and figures. (ZIP) [file pone.0305115.s001.zip › Supplementary explanations.pdf]

Nuclear Energy Acceptance in Poland: From Societal Attitudes to Effective Policy Strategies - Network Modeling Approach

Supplementary explanations

Ising model

Ising model estimation

We estimated the Ising model using regularized logistic regression with model selection based on the Extended Bayesian Information Criterion (EBIC) algorithm developed by van Borkulo et al. (2014) and implemented in the IsingFit package in R (https://CRAN.R-project.org/package=IsingFit). The model was regularized using the graphical lasso method (Friedman et al., 2008), which selects edges with the highest probability of occurrence in the true population and provides an alternative to significance tests for complex statistical models. If an edge is not significant (i.e. two beliefs do not align in the true population), the lasso method reduces the value of this edge to zero, and it is not represented in the graph. Although the lasso method allows significance analysis for complex models like networks, this comes at the expense of quantitative interpretation of the coefficients. The absolute coefficient values obtained by the lasso method no longer have quantitative meaning like classical regression coefficients. However, it is still possible to interpret their relative magnitudes. Coefficients with the largest absolute values reflect the relationships with the largest effect size in the true population (Hastie et al., 2009).

Ising model interpretation

The Ising model generates a network weights matrix that represents the relationships between various beliefs, attitudes, and political ideologies related to topics like nuclear energy, renewable energy, and climate change. This matrix is then visualized as a network graph, providing a clear illustration of these complex relationships.

Network graph components

In the graph, **nodes** represent individual beliefs or attitudes, while **edges** signify relationships between them. There are two types of relationships:

- **Alignment:** When two beliefs share the same value (+1 or -1), indicating a positive correlation. This is represented by a **blue edge**.
- **Divergence:** When two beliefs have opposing values (+1 and -1), indicating a negative correlation. This is represented by a **red edge**.

Alignment examples

- If ”Nuclear Energy is a good investment” and ”Nuclear Energy is safe” both have a value of +1, a thick blue edge connects them, indicating that individuals who hold one of these beliefs are likely to hold the other as well.
- Similarly, if ”Nuclear energy is a bad investment” and ”Nuclear energy is not safe” both have a value of -1, they are also connected by a blue edge, indicating alignment.

Divergence examples

- If ”Liberal political ideology” has a value of +1 and ”Nuclear Energy is a good investment” has a value of -1, a red edge connects them, indicating that individuals who identify as liberal tend to believe nuclear energy is a bad investment, while conservatives tend to believe it’s a good investment.

- Similarly, if ”Support for renewable energy” has a value of +1 and ”Nuclear energy is a good investment” has a value of -1, a red edge connects them, indicating divergence in beliefs.

Data analysis

Software version requirement for IsingFit

For our analysis, we used version 0.3.0 of the `IsingFit` software package. It is important to use this specific version because, in versions 0.4.0, a key feature for estimating -1 and +1 values was temporarily depreciated. To fully reproduce our study's results, you'll need to either:

1. Install version 0.3.0 of the `IsingFit` package, which can be done by running the following command in R:

```
# Install specific version of IsingFit used for analysis
devtools::install_version("IsingFit", version = "0.3.0")
```

2. Use a future version of `IsingFit` that re-enables the -1 and +1 estimation capability.

Code breakdown

Readers are assumed to have the required packages installed, including but not limited to, the necessary libraries and software dependencies, to ensure a seamless execution of the instructions and code provided.

Listing 1: Loading necessary libraries

```
library(qgraph)
library(IsingFit)
```

This part of the code loads the required libraries. The `qgraph` library is utilized for network visualizations, whereas the `IsingFit` library is used to fit the Ising model.

Listing 2: Assigning data

```
data <- #Here load the dataset from the OSF
```

This line of code assigns a new name to the downloaded dataset.

Listing 3: Fitting the Ising Model with regularized logistic regression

```
IsingModel <- IsingFit(data[1:22], plot = F)
```

In this segment, the code fits the Ising model to the first 22 columns of the `data` dataset using regularized logistic regression. Specifically, model selection is based on the Extended Bayesian Information Criterion (EBIC) algorithm developed by van Borkulo et al. (2014). This procedure is seamlessly integrated within the 'IsingFit' package in R.

Listing 4: Grouping variables

```
groups_list <- list(General = (1:6),
                    Renewable = (7:14),
                    Nuclear = (15:22))
```

This code creates a list, `groups_list`, which defines three groups: General, Renewable, and Nuclear. These groups represent ranges of columns in the dataset.

Listing 5: Defining labels

```
labs <- c(      "Political",
                "Urgency",
                ...
                "Prices",
                "Investment")
```

This segment of the code creates a vector, `labs`, which holds the labels for the nodes in the graph. The readers are free to specify this vector as they see fit. Our specification can be found in Tables 1 and 2, as well as Figure 1 of the manuscript.

Listing 6: Generating the network visualization for Figure 1

```
qgraph(IsingModel$weiadj, groups = groups_list, layout = 'groups',
       colors = c('#E9C46A','palegreen3','#00B0F0'),legend = F,
       labels = labs, label.cex = 1.15,
       node.width = 1.3,
       theme = 'colorblind')
```

The `qgraph` function produces the network model showcased as Figure 1 in our article. The parameters dictate the visualization's characteristics, such as node groupings, color themes, labels, and additional aesthetic properties.

Inspecting model weight

Listing 7: Displaying the weight matrix

```
View(IsingModel$weiadj)
```

This line of code displays the weight matrix for the Ising Model, representing the connections between the variables. The weight matrix, `IsingModel$weiadj`, contains the estimated edge weights between the nodes in the network. If the weight between two nodes is larger than 0, it means that the connection is significant according to the lasso method.

For example, consider the weight between the "Political Orientation" node (with liberal labeled as +1 and conservative labeled as -1) and the "Nuclear: investment attitude" node (with good investment labeled as +1 and bad investment labeled as -1). The weighted edge of -0.487 between these two nodes represents a divergence of liberal beliefs with a positive attitude towards nuclear investment, and conversely, an alignment of conservative attitudes with a positive attitude towards investment in nuclear energy. The weight coefficient is similar to a Chi-square test for independence, but more precise.

If the weight coefficient is present (significant), the Chi-square test between these two beliefs will always be significant. In the case of the Chi-square test for political orientation and nuclear investment attitude, the Chi-square statistic is significant below p<0.0001 However, the converse is not always true. If a Chi-square test is significant, the Ising model coefficient does not have to be significant, because the Ising model takes into account all other connections and hence is more precise in inferring the dependence between attitudes and beliefs.

References

Friedman, J., Hastie, T., and Tibshirani, R. (2008). Sparse inverse covariance estimation with the graphical lasso. *Biostatistics*, 9:432–441.

Hastie, T., Tibshirani, R., Friedman, J. H., and Friedman, J. H. (2009). *The elements of statistical learning: data mining, inference, and prediction.* Springer, New York.

van Borkulo, C. D., Borsboom, D., Epskamp, S., Blanken, T. F., Boschloo, L., Schoevers, R. A., and Waldorp, L. J. (2014). A new method for constructing networks from binary data. *Scientific Reports*, 4:5918.
