# Supplementary material for: Nuclear energy acceptance in Poland: From societal attitudes to effective policy strategies—Network modeling approach
Source: PLoS One. 2024 Aug 2;19(8):e0305115. doi: 10.1371/journal.pone.0305115 (PMC11296647; doi:10.1371/journal.pone.0305115)
Supplement: S1 File — The supplementary materials include: Dataset: This contains the raw data used in the analysis. Supplementary explanations: This document provides a breakdown of the code and additional explanations related to the model used in the analysis. Supplementary tables: This document contains supplementary tables with additional data and information related to Figs 2 and 3 in the main text. R script: This is the R script containing the code used for data analysis and generating the results and figures. (ZIP) [file pone.0305115.s001.zip › Supplementary tables.pdf]

Tables for Figure 1 and 2

The next eight tables display the frequencies and percentages corresponding to Figures 1 and 2. These tables present the data in a numerical format, allowing for a closer examination of the findings.

Figure 2.1: Contingency Table for Nuclear: Energy Independence

Political Orientation	Nuclear Risk Trade-off	Decreases Independence (%)	Increases Independence (%)
Conservative	Negative	22 (6.51%)	93 (27.5%)
Conservative	Positive	5 (1.48%)	24 (7.10%)
Liberal	Negative	13 (3.85%)	43 (12.7%)
Liberal	Positive	46 (13.6%)	92 (27.2%)

Figure 2.2: Contingency Table for Conventional Energy Attitude

Political Orientation	Nuclear Risk Trade-off	Decreases Energy Use (%)	Increases Energy Use (%)
Conservative	Negative	96 (28.4%)	19 (5.62%)
Conservative	Positive	20 (5.92%)	9 (2.66%)
Liberal	Negative	23 (6.80%)	33 (9.76%)
Liberal	Positive	28 (8.28%)	110 (32.54%)

Figure 2.3: Contingency Table for Nuclear: Operation Safety

**Political Orientation**	**Nuclear Risk Trade-off**	**Unsafe (%)**	**Safe (%)**
Conservative	Negative	18 (5.33%)	97 (28.7%)
Conservative	Positive	3 (0.888%)	26 (7.69%)
Liberal	Negative	15 (4.44%)	41 (12.1%)
Liberal	Positive	26 (7.69%)	112 (33.1%)

Figure 2.4: Contingency Table for Nuclear: Waste Safety

**Political Orientation**	**Nuclear Risk Trade-off**	**Unsafe (%)**	**Safe (%)**
Conservative	Negative	37 (10.9%)	78 (23.1%)
Conservative	Positive	13 (3.85%)	16 (4.73%)
Liberal	Negative	35 (10.4%)	21 (6.21%)
Liberal	Positive	111 (32.8%)	27 (7.99%)

Figure 3.1: Contingency Table for Nuclear: Efficiency

**Political Orientation**	**Nuclear Investment Attitude**	**Inefficient (%)**	**Efficient (%)**
Liberal	Negative	4 (1.18%)	25 (7.40%)
Liberal	Positive	11 (3.25%)	104 (30.8%)
Conservative	Negative	43 (12.7%)	60 (17.8%)
Conservative	Positive	32 (9.47%)	59 (17.5%)

Figure 3.2: Contingency Table for Conventional Energy Attitude

**Political Orientation**	**Nuclear Investment Attitude**	**Continue (%)**	**Phase Out (%)**
Liberal	Negative	23 (6.80%)	6 (1.78%)
Liberal	Positive	93 (27.5%)	22 (6.51%)
Conservative	Negative	26 (7.69%)	77 (22.8%)
Conservative	Positive	25 (7.40%)	66 (19.5%)

Figure 3.3: Contingency Table for Nuclear: Prices

**Political Orientation**	**Nuclear Investment Attitude**	**Price Increase (%)**	**Price Decrease (%)**
Liberal	Negative	11 (3.25%)	18 (5.33%)
Liberal	Positive	49 (14.5%)	66 (19.5%)
Conservative	Negative	35 (10.4%)	68 (20.1%)
Conservative	Positive	27 (7.99%)	64 (18.9%)

Figure 3.4: Contingency Table for Nuclear: Waste Safety

**Political Orientation**	**Nuclear Investment Attitude**	**Unsafe (%)**	**Safe (%)**
Liberal	Negative	8 (2.37%)	21 (6.21%)
Liberal	Positive	42 (12.4%)	73 (21.6%)
Conservative	Negative	73 (21.6%)	30 (8.88%)
Conservative	Positive	73 (21.6%)	18 (5.33%)

Additional tables

Political Comparison Tables: Tables 1-9 stratify responses to nuclear energy questions by self-reported ideology. The responses between ideologies are compared statistically through the use of Chi-Squared tests with reported P values. Response percentages and counts (N) are provided.

Table 1: Attitude Towards Nuclear as an Investment – $p < .0001$

**Political Orientation**	**Bad Investment (%)**	**Good Investment (%)**
Conservative	20.14% (29)	79.86% (115)
Liberal	53.09% (103)	46.91% (91)

Table 2: Climate Countermeasures Opinion – $p < .0001$

**Political Orientation**	**No Climate Countermeasures (%)**	**Climate Immediate Action (%)**
Conservative	87.50% (126)	12.50% (18)
Liberal	11.86% (23)	88.14% (171)

Table 3: Conventional Energy Investment – $p < .0001$

**Political Orientation**	**Invest in Conventional Energy (%)**	**Phase Out Conventional (%)**
Conservative	80.56% (116)	19.44% (28)
Liberal	26.29% (51)	73.71% (143)

Table 4: Environmental Utilization vs. Preservation – $p < .0001$

Political Orientation	Utilize Environment (%)	Preserve Environment (%)
Conservative	84.03% (121)	15.97% (23)
Liberal	22.16% (43)	77.83% (151)

Table 5: State Support for Energy Transition – $p < .0001$

Political Orientation	State Does Not Support Transition (%)	State Supports Transition (%)
Conservative	77.08% (111)	22.92% (33)
Liberal	30.41% (59)	69.59% (135)

Table 6: Energy Market Decentralization – $p < .0001$

Political Orientation	Energy Market Decentralized (%)	Energy Market Centralized (%)
Conservative	79.86% (115)	20.14% (29)
Liberal	39.18% (76)	60.82% (118)

Table 7: Nuclear Risks vs. Benefits – $p < .0001$

Political Orientation	Nuclear Risks Outweigh Benefits (%)	Nuclear Benefits Outweigh Risks (%)
Conservative	79.86% (115)	20.14% (29)
Liberal	28.87% (56)	71.13% (138)

Table 8: Efficiency of Nuclear Energy – $p < .0001$

Political Orientation	Nuclear is Not Efficient (%)	Nuclear is Efficient (%)
Conservative	10.42% (15)	89.58% (129)
Liberal	38.66% (75)	61.34% (119)

Table 9: Safety of Nuclear Waste – $p < .0001$

Political Orientation	Nuclear Waste Not Safe (%)	Nuclear Waste is Safe (%)
Conservative	34.72% (50)	65.28% (94)
Liberal	75.26% (146)	24.74% (48)
