# Supplementary material for: Nuclear energy acceptance in Poland: From societal attitudes to effective policy strategies—Network modeling approach
Source: PLoS One. 2024 Aug 2;19(8):e0305115. doi: 10.1371/journal.pone.0305115 (PMC11296647; doi:10.1371/journal.pone.0305115)
Supplement: Supplementary file 2 [file pone.0305115.s002.docx]

**Ref: PONE-D-23-38813**

**Title: Nuclear Energy Acceptance in Poland: From Societal Attitudes to Effective Policy Strategies - Network Modeling Approach**

**Journal: PLOS ONE**

We sincerely thank the Editor for all comments regarding our manuscript. We have made a concerted effort to meticulously address the feedback on formatting, as well as any formal oversights in the submitted text. We have also checked and supplemented the links and additional material as required. We hope that our revisions have met expectations and that our paper is now suitable for publication. Thank you for providing us with the opportunity to refine our work. Below, we present a summary of each comment received along with our respective responses.

**Editor comments:**

1. When submitting your revision, we need you to address these additional requirements. Please ensure that your manuscript meets PLOS ONE's style requirements, including those for file naming. The PLOS ONE style templates can be found at
   <https://journals.plos.org/plosone/s/file?id=wjVg/PLOSOne_formatting_sample_main_body.pdf> and <https://journals.plos.org/plosone/s/file?id=ba62/PLOSOne_formatting_sample_title_authors_affiliations.pdf>

**Response E1:** *Thank you for your guidance on the formatting requirements. We have thoroughly reviewed the PLOS ONE style templates and have adjusted our manuscript accordingly to meet the guidelines provided, including those for file naming.*

2. Please update your submission to use the PLOS LaTeX template. The template and more information on our requirements for LaTeX submissions can be found at <http://journals.plos.org/plosone/s/latex>.

**Response E2:** *We* *have updated our manuscript using the provided PLOS LaTeX template as specified and have ensured compliance with all the guidelines outlined.*

3. Did you know that depositing data in a repository is associated with up to a 25% citation advantage (<https://doi.org/10.1371/journal.pone.0230416>)? If you’ve not already done so, consider depositing your raw data in a repository to ensure your work is read, appreciated and cited by the largest possible audience. You’ll also earn an Accessible Data icon on your published paper if you deposit your data in any participating repository (<https://plos.org/open-science/open-data/#accessible-data>).

**Response E3:** *Thank you for emphasizing the benefits of depositing our data in a repository. We share the same views and have indeed linked our data repository to our paper, ensuring it is accessible for verification and reuse. Our dataset is deposited with OSF, which is on the recommended repository list by the journal. The current link to our repository is:* [*https://osf.io/26n4u/?view_only=8d32a81d03354735832c2068a715d663*](https://osf.io/26n4u/?view_only=8d32a81d03354735832c2068a715d663)*.
This link is currently set to a "view-only" mode for review purposes to maintain anonymity during the peer review process. Upon acceptance of our paper, we will update the link to a more concise and publicly accessible format.*

4. You indicated that ethical approval was not necessary for your study. We understand that the framework for ethical oversight requirements for studies of this type may differ depending on the setting and we would appreciate some further clarification regarding your research. Could you please provide further details on why your study is exempt from the need for approval and confirmation from your institutional review board or research ethics committee (e.g., in the form of a letter or email correspondence) that ethics review was not necessary for this study? Please include a copy of the correspondence as an ""Other"" file.

**Response E4:** *In December 2022, before starting the study, we submitted an electronic application to the Research Ethics Committee of the University of Gdańsk, presenting the purpose and scope of the planned survey research. The application was approved by the Commission in the application system. In the review, the editor asked us for clarification and opinion on this matter. We asked the Ethics Committee for such an opinion and we attached it to the correspondence.*

5. Thank you for stating the following financial disclosure: This research was funded by the University of Gdansk, Faculty of Economics. Internal financing. Please state what role the funders took in the study. If the funders had no role, please state: "The funders had no role in study design, data collection and analysis, decision to publish, or preparation of the manuscript." If this statement is not correct you must amend it as needed. Please include this amended Role of Funder statement in your cover letter; we will change the online submission form on your behalf.

**Response E5:**  *Thank you for your instructions regarding the financial disclosure statement. We confirm that the founder, the University of Gdansk, Faculty of Economics, had a role in data collection. We have included the amended "Role of Funder" statement in our cover letter. Please change on our behalf this amended Role of Funder statement the online submission form.*

6. When completing the data availability statement of the submission form, you indicated that you will make your data available on acceptance. We strongly recommend all authors decide on a data sharing plan before acceptance, as the process can be lengthy and hold up publication timelines. Please note that, though access restrictions are acceptable now, your entire data will need to be made freely accessible if your manuscript is accepted for publication. This policy applies to all data except where public deposition would breach compliance with the protocol approved by your research ethics board. If you are unable to adhere to our open data policy, please kindly revise your statement to explain your reasoning and we will seek the editor's input on an exemption. Please be assured that, once you have provided your new statement, the assessment of your exemption will not hold up the peer review process.

**Response E6:** *Our data can be accessed at the OSF repository using this link:* [*https://osf.io/26n4u/?view_only=8d32a81d03354735832c2068a715d663*](https://osf.io/26n4u/?view_only=8d32a81d03354735832c2068a715d663)*, which is currently in "view-only" mode for review purposes.*

*We are open to sharing and storing our data directly on the journal's website, should this be preferred. We are more than willing to comply with any arrangements that facilitate easier access to our data and adhere to your publication's policies. Please let us know if further adjustments are required or if there are other preferred options for data deposition than OSF repository.*

7. We are unable to open your Supporting Information file figure1.svg. Please kindly revise as necessary and re-upload.

**Response E7:** *Thank you for notifying us about the issue with the figure1 file. We have revised and reuploaded all figures, including figure1, in *.tiff format as required.*

8. Please include captions for your Supporting Information files at the end of your manuscript, and update any in-text citations to match accordingly. Please see our Supporting Information guidelines for more information: <http://journals.plos.org/plosone/s/supporting-information>.

**Response E8:** *In our manuscript, the Supporting Information is currently consolidated into a single link to our OSF repository, where readers can access the data, code, code explanations, and additional tables associated with our study. We have adhered to the guidelines provided in the PLOS LaTeX template for referencing this link in the manuscript. Consequently, we do not use any captions within the text for Supplementary Materials beyond referring to the repository link itself.*

*We are open to storing our data directly on the journal’s website, should this be an option. If the journal supports hosting our data, we would then use separate captions for data, tables, and code breakdown as part of the Supporting Information, according to the journal's specifications. Please advise us on the feasibility of hosting the data on the journal's platform, and let us know if any further modifications are necessary.*

**Reviewer #1**:  We sincerely thank Reviewer #1 for their insightful comments and guidance, which have been invaluable in enhancing our manuscript and addressing a few issues. We have carefully considered and responded to all the feedback provided during the review process. We hope that our revisions meet Reviewer #1's expectations and that our paper is now ready for publication. Thank you for the opportunity to improve our work. Below, we have detailed each comment along with our corresponding responses.

**Comment #1.1**  I would recommend that, for a better understanding, include a simple example of causal attitude networks. That would make it easier for the readers to follow.

**Response to comment #1.1:** *Thank you for the suggestion to include a simple example of causal attitude networks. In response, we have extended the example of the Ising model as a causal attitude network in the “Network Model” section of the main text, as well as provided even more detailed explanations within the Supplementary explanation PDF, which can be accessed through the OSF link in the Supporting Information section. We hope that these changes, including the expanded examples, will aid readers in better understanding our results. We appreciate your feedback.*

**Comment #1.2:** Page 17, rather than the term "creation" for Nuclear power plant, My suggestion is to use "construction".

**Response to comment #1.2:** *We have changed the term “creation” to “construction” as suggested.*

***Reviewer #2:*** We sincerely thank Reviewer #2 for their insightful comments and guidance, which have been invaluable in enhancing our manuscript and addressing several critical issues. We have carefully considered and responded to all the feedback provided during the review process. We hope that our revisions meet Reviewer #2's expectations and that our paper is now ready for publication. Thank you for the opportunity to improve our work. Below, we have detailed each comment along with our corresponding responses.

**Comment #2.1:**
• The article adheres to appropriate reporting guidelines and standards for data availability.
• The authors state the survey data and R code are accessible via a provided link. However, permissions are required to access the data.

**Response to comment #2.1:**

*Thank you for your comment regarding the data availability. We understand the importance of data transparency and accessibility in scientific research, and we have made efforts to ensure that our materials are readily available. Regarding the provided link for accessing the survey data and R code, we apologize for the inconvenience you experienced. It is indeed odd that the anonymized link did not allow you to access the data, as we have tested it, and it should have worked without requiring any permissions or a registered account.*

*We regenerated the link and the current anonymized link should work without the need for permissions:*
[*https://osf.io/26n4u/?view_only=8d32a81d03354735832c2068a715d663*](https://osf.io/26n4u/?view_only=8d32a81d03354735832c2068a715d663)*.*

*Additionally, we have extensively updated our supplementary materials to include more than just the code and dataset. The supplementary materials now include the following:*

- *Dataset*
- *Supplementary explanations which provide a breakdown of the code and additional explanations related to the model used in the analysis.*
- *Supplementary tables with additional data and information related to Figures 2 and 3 in the main text.*
- *R script containing the code used for data analysis and generating the results and figures.*

*We have provided the link once more, and we sincerely hope that it will work without any issues this time. If the problem persists, we will gladly provide the supplementary data directly to the editor, who can then forward it to you.*

**Comment #2.2:** I am a bit concerned to see that institutional ethical review was not conducted for the survey questions and approach. I would like the authors to elaborate the university standard policies on this.

**Response to comment #2.2:** *In December 2022, before starting the study, we submitted an electronic application to the Research Ethics Committee of the University of Gdańsk, presenting the purpose and scope of the planned survey research. The application was approved by the Commission in the application system.*

**Comment #2.3:** Figure and Table captions – I cannot find figure and table captions in the review material. These must be provided.

**Response to comment #2.3:** *Thank you for pointing out the omission of figure and table captions in our initial submission. This was an oversight on our part. We have corrected this error in the revised submission, and all figures and tables now include appropriate captions as per the journal's guidelines.*

**Comment #2.4:**

- Table 1 – the information content is confusing. Specifically the information presented on “proportion supporting” need to be elaborated in the text or in explanatory material associated with the table.
- Figures 2 and 3 – I appreciate the author’s effort to show 3 dimensions of information in 2D figures. However, the information took me a while to understand, particularly with figure captions available. The most important issues to address is for the “+” and “-“ symbols. Although size is meant to represent response rate, I need to also see the specific response rate numbers. This should at least be available in supplementary material.

**Response to comment #2.4:** *Thank you for your feedback regarding Table 1 and Figures 2 and 3. We have made the following revisions to address the issues raised:*

*Table 1: We have revised the text within the manuscript to clarify the "proportion supporting" column, which represents the percentage of respondents who hold a supporting belief (second column) for each construct listed in the table. We have added notes associated with the table to better guide readers through the presented data.*

*Figures 2 and 3: The "+" and "-" symbols in Figures 2 and 3 are meant to give readers a quick and easy way to see the key differences and comparisons in the data at a glance. Alongside these symbols, percentage data are directly displayed on the figures to provide an immediate quantitative context which complements the symbolic representation. That said, we have added the specific response rate numbers to the supplementary materials file titled "Supplementary tables.pdf". This file provides contingency tables of frequencies that correspond to the symbols used in the figures. We believe combining symbolic visuals and detailed supplementary data provides the best of both worlds - an at-a-glance overview for those who want it, plus granular data for closer analysis.*

*Additionally, we have revised the figure captions to improve clarity. The captions now explain that the percentages for each part of the figures sum up to 100%, accounting for any rounding errors. We have also modified the numbering in the figure captions from previous formats like "1)" to numerical labels we use as references in text such as "Figure 2.1" or Figure 3.1".*

**Comment #2.5:** Statistical analyses – perhaps the most important issue to address, and the cause for requested major revision, is statistical analysis/confirmation of differences between survey response rates. Figures 2 and 3 provide a number of response rates but without any information regarding true statistical differences. Appropriate statistical tests are needed in supplementary material for all cases when comparisons are being made concerning response rates. As an example, the statement on page 22 “most Liberals do see nuclear energy as efficient enough to supply most of a nation’s energy [3.1] and believe that nuclear energy will decrease energy prices if introduced [3.3].” cannot be verified given the information provided currently. Similarly, on page 30 I see the statement “Similarly, most Liberal respondents do not support continued investment into nuclear energy. However, they see nuclear energy as efficient and believe it will decrease energy prices.” However, in Figure 3 I question whether “most Liberal respondents do not support continued investment into nuclear energy” is a valid statement statistically. I need to see the data.

**Response to comment #2.5:** *Thank you for your valuable feedback. We acknowledge that our initial phrasing of the effects observed in our data was imprecise. We have revised our statements to better reflect the statistical analyses conducted and the relationships identified in our study.*

*We primarily focus on the dependence of various attitudes and beliefs using the network model and network weights. However, in the section "Granular Level Results," our aim was to highlight different attitudes associated with the central belief identified in our analysis - political orientation - and significant attitudes such as investment attitude and risk-benefit trade-off. We have completely revamped this section and the statements presented there. For example, the statement "most Liberal respondents do not support continued investment into nuclear energy," which was imprecise as it concerned proportions, has been rephrased to "In contrast to Liberals, Conservative respondents significantly support increased investment in nuclear energy," which concerns association.*

*In addition, we have added tables with chi-squared test results in the supplementary materials for readers who may not be as familiar with the network model (model weights are also available in the supplementary material through the provided code). New tables provide response percentages and counts and chi-squared p-values supporting our claims.*

*We have also included a brief discussion on the relationships observed, using them to explain the patterns identified, while the main discussion remains in the next section.*

*We want our paper to be accessible to a broad audience, which is why we moved many detailed statistical measures to the supplementary materials. We hope the reviewers understand our choice and find the revised presentation more clear and effective.*

**Reviewer #3:** We are truly grateful to Reviewer #3 for their detailed comments and valuable guidance, which have significantly contributed to the improvement of our manuscript and the resolution of important issues. We have thoroughly addressed all the feedback received throughout the review process. We hope that our revisions have met Reviewer #3's expectations and that our paper is now suitable for publication. Thank you for providing us with the opportunity to refine our work. Below, we present a summary of each comment received along with our respective responses.

**Comment #3.1:** Paragraph 30 of the conclusion mentions that ’Conservatives, on the other hand, generally support continued investment into nuclear energy.’ The conclusions are overstated. Please explain the meaning of ‘Conservatives’.

**Response to Comment #3.1:** *We appreciate the reviewer's comment regarding our meaning of conservatives. As noted in the revised 'Conceptualization of Attitudes' section, our study defined political ideology through self-report measures, which is a standard approach used in prior research on this topic (see references 10 to 13 from ‘Political Ideology’ section).*

*Respondents were asked to self-identify as liberal/left-leaning or conservative/right-leaning. Our data clearly shows that among those self-identifying as conservative, 80% supported continued investment in expanding nuclear energy's share (see Figure 3 and newly added contingency tables in Supplementary materials). This aligns with findings from previous studies that have found a correlation between conservative self-reported ideology and greater acceptance of nuclear power across various countries (e.g., Chung and Kim, 2018). In the Polish sample, self-identified conservatives were significantly more supportive of nuclear investment compared to liberals (see network model and Supplementary materials for detailed significance inference). This finding is not overstated and highlights an ideological divide that should be acknowledged in the conclusions.*

**Comment #3.2:**  In section 2.4 , ’ found that many anti-nuclear protesters worldwide primarily highlight issues related to waste management and environmental pollution’ and ’the emphasis on risk and safety might not be as influential in determining public acceptance as previously believed.’ Why the emphasis is ‘not’ be influential?

**Response to Comment #3.2:** *Thank you for pointing out the unclear argument in section “Risk Perception and Safety Concerns”. We meant to convey that a previous study by Pidgeon et al. (2008) has stated that the emphasis on “operation” risks and safety (i.e., campaigns that focus on reassuring the public that another Chernobyl will not happen) might not be as effective in garnering support for nuclear energy, because the public's concerns are more focused on long-term environmental impacts, as well as waste management issues. We have revised the section to clarify the reasoning behind the statement. The revised text now reads: “Pidgeon et al. \cite{pidgeon2008} suggest that the emphasis on power plant operation safety might not be as influential in determining public acceptance as previously believed, because the public's concerns about nuclear energy are more focused on the long-term environmental impacts, rather than operation risks and safety issues.”. We hope this revised argument is now clear and addresses your concern.*

**Comment #3.3:** Please explain the meaning of ‘the lens of investment attitude and perceived advantageous trade-off.’

**Response to Comment #3.3:** *We have added additional language to clarify the meaning of the ‘investment’ and ‘tradeoff’. The section now reads: “Specifically, we measure public acceptance through the lens of investment attitude and perceived advantageous trade-off. We ask respondents whether they think expanding the share of nuclear energy in the energy mix is a good investment and whether the potential benefits of nuclear energy outweigh associated risks. We use these two questions as proxies for the acceptance of nuclear energy and determine what other beliefs are associated with respondent answers to the two questions. “*

**Comment #3.4:** Please consider to add some reference about the design of new generation nuclear reactor.

**Response to Comment #3.4:** *Thank you for the valuable suggestion to include references on the design of next-generation nuclear reactors. We appreciate you bringing these important works to our attention. As per your recommendation, we have incorporated these references in the "Economic Considerations" subsection of the Literature Review. The references highlight the ongoing research efforts aimed at optimizing the safety, efficiency, and sustainability aspects of advanced nuclear reactor designs. These technological advancements could potentially influence the economic viability and public perception of nuclear energy in the future. We are grateful for your feedback, as it has allowed us to provide a more comprehensive overview of the current landscape surrounding nuclear energy acceptance.*

**Reviewer #4:** We greatly appreciate Reviewer #4 for their comprehensive feedback and expert advice, which were crucial in refining our manuscript and resolving key issues. We trust that our revisions align with Reviewer #4's expectations and that our manuscript is now prepared for publication. Below, each comment from the review is outlined along with our corresponding responses.

**Comment #4.1:** page 3: "typical lifespan of a nuclear power plant, which could exceed 80 years." is more appropriate since no plant have yet obtained a license to operate more than 80 years (even if this is considered).

**Response to Comment #4.1:** *We have modified the wording as suggested from ‘can’ to ‘could’.*

**Comment #4.2:** page 4: "we consider factors like political ideology, safety concerns" as well as "environmental and economic variables."

**Response to Comment #4.2:** *We have modified the wording to state: “we consider factors such as political ideology and safety concerns as well as environmental and economic variables”.*

**Comment #4.3:** page 8:"Recent surveys conducted by CBOS" indicate what CBOS is when first used in the text.

**Response to Comment #4.3:** *Thank you for pointing that out. In response, we have clarified the introduction of this research agency in the text. The revised sentence will read as follows:*

*"Recent surveys conducted by the Polish Centre for Public Opinion Research (CBOS, Polish: Centrum Badania Opinii Społecznej)..." In subsequent mentions, we will use the abbreviation CBOS.*

**Comment #4.4:** page 19: "with respect to both" and "nuclear and renewable energy" (and to be deleted)

**Response to Comment #4.4:** *We appreciate the suggestion. We have deleted the extraneous “and’”.*

**Comment #4.5;** page 19 and Figure 1: Figure caption not provided on the PLOS ONE page. Is it included in the article. Figure caption and brief description of the figure, in the text, is needed.

**Response to Comment #4.5:** *Thank you for pointing out the omission of figure and table captions in our initial submission. This was an oversight on our part. We have corrected this error in the revised submission, and all figures and tables now include appropriate captions as per the journal's guidelines.*

**Comment #4.6:** page 20: "Political ideology was found to be the most central determinant in predicting respondent stances on acceptance of energy sources and many other beliefs." is the main conclusion of paper. However, according to the data provided, 144 respondents defined themselves as conservative, out of 338 respondents. This corresponds to 42.6 % of the respondents. This value should be mentioned in the paper and commented. Is this proportion in agreement with the proportion of conservatives in Poland at the time of the survey? In other terms is the sample representative? Potentially, the perception of being conservative or liberal may not be related to politics (and political leaders) but to a personal definition of the respondents. What would be the impact on the conclusions of the paper?

**Response to Comment #4.6:**  *We acknowledge that self-identifying as conservative or liberal may not necessarily correspond to affiliations with contemporary political parties. Instead, we utilize these terms to denote ideologies or ideological groups, rather than as direct representations of specific political affiliations. This approach is deemed more suitable given that political parties can evolve significantly over time, whereas ideologies tend to remain more stable. It is common in attitude research to categorize respondents into ideological groups based on their self-reported beliefs, as evidenced by previous studies exploring the relationship between political ideology and attitudes towards nuclear energy ("Recent surveys conducted by the Polish Centre for Public Opinion Research (CBOS) in 2021).*

**Comment #4.7** page 21: The analysis in section 7.2 contains a number of redundancies. Indeed, the paragraphs "conservatives" and "liberals" include comparisons which are also addressed in the paragraph "political comparison". This section should be revised to suppress these redundancies.

**Response to Comment #4.7:** *We agree that repeating the comparisons is both redundant and unhelpful. This section has been completely revamped, and there are no redundant sections now. The “political comparisons” section has been incorporated into the one section titled: Granular Level Results: Conservatives vs Liberals.*

**Comment #4.8:** page 24: "These two" latter "pathways likely explain why political ideology is such a strong indicator of nuclear acceptance views." (add latter)

**Response to Comment #4.8:** *We have modified the text as suggested.*
